# Focused Cardiac Assessment in Kidney Care

**DOI:** 10.24908/pocus.v7iKidney.14996

**Published:** 2022-02-01

**Authors:** Vineet Veitla, Bhavna Bhasin

**Affiliations:** 1 Division of Nephrology, Medical College of Wisconsin Milwaukee, WI

**Keywords:** FoCUS, POCUS, echocardiography, nephrology, hypotension, volume assessment, dyspnea

## Background

Point of care ultrasonography (POCUS) is considered to be a very useful and informative extension of the bedside physical exam. The information obtained from POCUS allows for real time assessment for expedited decision making to improve efficiency in patient care and management. Many programs across the country are now incorporating POCUS into their training schedules to allow their residents, fellows, and faculty to gain competence in the techniques and varied clinical uses of POCUS 1, 2, 3. In nephrology, POCUS has been used at the bedside for access planning, dialysis catheter placement, and to guide kidney biopsies to mention a few applications 4. There is a wide scope for POCUS in nephrology in addition to kidney and bladder assessment. This includes focused cardiac ultrasound to evaluate the heart for structural and functional abnormalities and lung ultrasound as well. These bedside ultrasound assessments help with point of care management decisions pertaining to volume assessment in acute and chronic kidney disease, adjustment of ultrafiltration goals in dialysis patients, and evaluation of hypotension and dyspnea.

This review describes the techniques, interpretation, and application of focused cardiac ultrasound for the nephrologist (FoCUS).

## Ultrasound Probe

A fundamental component of ultrasound imaging is choosing the right transducer or probe. The probe used for cardiac assessment is made from piezoelectric crystals which generate fine vibrations in a phenomenon called reverse piezoelectric effect 5. These vibrations are converted to ultrasonic waves that are transmitted to the tissue and then reflected back to the probe. This effect causes mechanical distortion of the crystals, which is converted to an electric current and then used by the ultrasound machine to generate an image 6.

**Transducer types: **Ultrasound transducers differ in construction based on piezoelectric crystal arrangement, aperture (footprint), and frequency. The transducer used for cardiac ultrasound is a phased array transducer which produces diverging low frequency ultrasound beams (1 to 5 MHz) that generate a pie shaped format with room for focus adjustment and steering. Differential excitation of the piezoelectric crystals creates rapid electronic beams by sequentially pulsing multiple small crystals within the transducer. This technology allows for efficient two dimensional imaging and is optimal for structures such as the heart. 

## Views in Cardiac Imaging 

**Figure 1:**
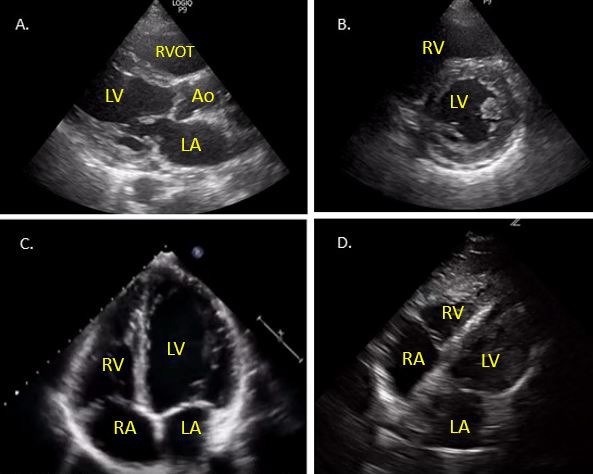
A) Parasternal long axis view. B) Parasternal short axis view. C) Apical 4-chamber view. D) Subcostal 4-chamber view. LA - left atrium, LV - left ventricle, RA - right atrium, RV - right ventricle, RVOT - right ventricular outflow tract,Ao - aorta.

Cardiac ultrasound requires assessment of both structure and function of the heart and the highest quality images should be acquired for optimal assessment. In order to be confident in the interpretation of cardiac ultrasound findings, views from multiple imaging planes should be obtained. Traditional teaching focuses on achieving proficiency in five core cardiac views: parasternal long axis (PLAX), parasternal short axis (mid ventricular level) (PSAX), apical 4-chamber (A4C), subcostal 4-chamber (S4C), and subcostal inferior vena cava (IVC) views 7. Images from these views are shown in Figure 1. 

## Imaging Windows and Views

### PARASTERNAL WINDOW

Traditionally cardiac ultrasound begins with the parasternal window. Ideally, the patient should be placed in a supine position, but if needed the patient can be made to lie in the left lateral decubitus position to bring the heart in direct contact with the chest wall.

**Parasternal long axis view (PLAX):** This is obtained by placing the phased-array probe immediately to the left of the sternum in the third or fourth intercostal space (ICS) with the probe marker pointing towards the right shoulder. The beam should be positioned parallel to a line running from the patient’s shoulder to the left hip. The appropriate window for imaging may lie anywhere between the second to the fifth intercostal spaces and sliding might help get the best images possible where the mitral valve should be positioned in the center of the ultrasound image. An ideal parasternal window has appropriate positioning of the cardiac structures and the least interference from the adjacent organs including the lung. Images obtained from this view demonstrate the anatomic cross-section of the long axis of the heart and include the aortic valve (AV), mitral valve (MV), left ventricle (LV), pericardium, right ventricular outflow tract (RVOT), left ventricular outflow tract (LVOT), and portions of the ascending and descending aorta (AO).

The right ventricle (RV) is visualized at the top of the screen. While keeping the probe steady, the mitral and the aortic valves can be seen and the ultrasound beam can be centered on the left ventricle. With slight rotation and tilting movements of the probe, the left ventricular cavity can be analyzed in its full extent. PLAX view is used primarily to assess LV size and function in addition to the AV, MV, and left atrial size. Circumferential pericardial effusions can also be detected in this view. However, assessment of right ventricle size and function is difficult from this view.

**Parasternal short axis views (PSAX): **An effective technique to acquire high-quality PSAX views is by starting with high-quality PLAX views. The probe is then rotated 90 degrees clockwise to point towards the patient left shoulder. Care must be taken not to allow the transducer to slide to a different position on the chest. The 2-hand maneuver can be tried so as to keep the transducer steady with one hand while the other hand rotates the transducer into position. While five different imaging planes can be achieved in the parasternal short axis view, the mid ventricular level is favored by most providers for its reliable portrayal of LV function. Mid-ventricular parasternal short axis view is achieved when both papillary muscles are visualized in cross-section and appear symmetric. The short axis mid-ventricular view is best for assessing global LV systolic function and segmental LV wall motion. This view also helps assess the shape and function of the interventricular septum in the setting of RV dilatation and dysfunction. Large or moderate sized circumferential pericardial effusions can also be visualized. 

**Pulmonary artery level:** From the mid-ventricular level the ultrasound beam is tilted towards the base of the heart. The plane is considered correct when the pulmonary valve (PV), main pulmonary artery (MPA), and AO are seen in short axis. In this view, pulmonary regurgitation velocities may be used to estimate the mean and diastolic pulmonary artery pressures.

**Figure 2:**
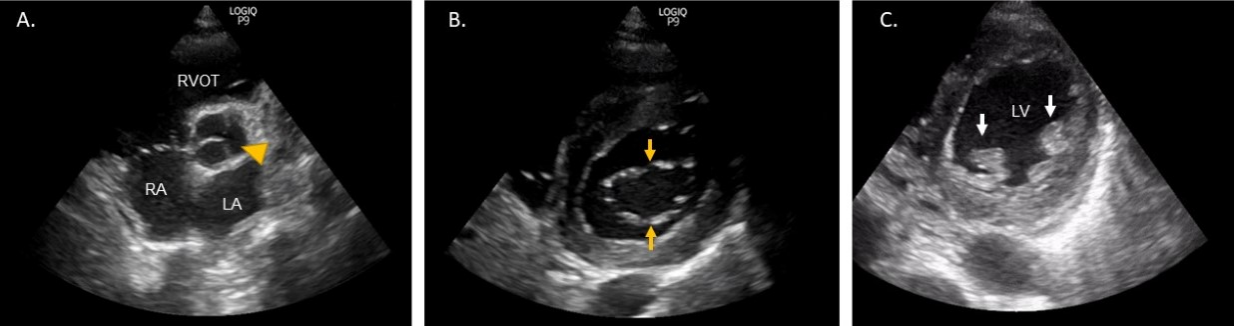
Parasternal short axis (PSAX) view at the level of A) aortic valve [arrowhead], B) mitral valve [arrows point to the valve leaflets], and C) papillary muscle/mid-ventricle [arrows point to papillary muscles]. RA: right atrium, LA: left atrium, RVOT: right ventricular outflow tract, LV: left ventricle.

**AV level:** From the pulmonary artery level, the transducer is tilted slightly inferiorly towards the apex of the heart. An ideal image includes short axis view of the AV, which may require a slight rotation of the transducer until all 3 AV cusps appear symmetric. This view allows assessment of AV and TV (Figure 2).

**MV level:** When rotating from parasternal long axis to short axis view, the distinct fish mouth appearance of the MV is usually seen first which allows for an assessment of the MV (Figure 2). 

**Mid-ventricular, papillary muscle level:** This view reveals the most useful clinical information in vast majority of acutely ill patients. Papillary muscles are symmetrically seen in cross-section in the center of the circular left-ventricular cavity. Motion of individual LV chamber wall segments is best assessed at this level, as well as overall LV systolic function (Figure 2).

**Apical level:** The short axis view is obtained by tilting the transducer to aim the ultrasound beam inferiorly, towards the apex of the heart. The left ventricular apex is visualized sequentially starting from the mid papillary muscle level and moving inferiorly. LV systolic function may be overestimated compared to the mid ventricular level. In rare cases an apical LV thrombus may be seen.

### APICAL WINDOW

Imaging window: The apical window usually follows the parasternal views. It is more difficult to obtain quality images from an apical window when compared to parasternal or subcostal windows. 

**Apical 4-Chamber View**

With the patient either in the left lateral decubitus position or supine with subtle leftward rotation, the probe is placed over the cardiac apex (inferolateral to the left nipple in males and below the inferolateral quadrant of the left breast in females). Once the apex is visualized, the transducer needs to be tilted steeply to aim the orientation marker to the patient’s left side. 

Once positioned in the apical area, the transducer should be rocked to align the interventricular septum in a vertical position in the center of the screen. The transducer might need to be rotated slightly so that the LV and RV cavities are viewed in a true longitudinal cross-section. In an ideal apical 4-chamber view the LV, RV, LA, and RA can all be seen clearly in addition to the MV and TV. The apical 4-chamber view helps in assessment of the RV and its systolic function. MV and TV can also be evaluated and pericardial fluid can be detected.

**Apical 2-Chamber View**

Starting from an apical 4-chamber view, the transducer is rotated 90 degrees counterclockwise. The ideal view reveals LV, LA, and MV. Under rotation or over rotation can reveal RV or LVOT respectively. This view allows assessment of the regional LV function.

**Apical 3-Chamber View**

Starting from an apical-2 chamber view, the transducer is further rotated 30 degrees counterclockwise. The ideal view demonstrates the LV, LA, and MV with the LVOT and AV appearing at the 5 o’clock position. Assessment of the inferolateral and anteroseptal LV walls can be ascertained from this position. 

### SUBCOSTAL WINDOW

In the subcostal view, the transducer is placed below the xiphoid process in the midline with the probe marker pointed to the patient left side. The ultrasound transducer face should be aimed upward towards the heart and left shoulder. Asking the patient to bend their knees to relax the abdomen or holding their breath momentarily at end of inspiration can transiently shift the heart downward into view. 

**Subcostal 4-Chamber View**

Once the heart is in view in the subcoastal window, subtle transducer manipulation can help in acquisition of an optimal 4-chamber view. Ideally the RV, LV, RA, LA, and pericardium should be visualized in cross section along the long axis of the heart. This view can help assess the RV free wall and perform a comparison of LV and RV size. However, as the imaging plane is through the inferior portion of the heart rather than the middle of the heart, RV size can be underestimated. This view also allows for visualization of pericardial effusion.

**Inferior Vena Cava View**

Once the traditional subcostal view is obtained, the transducer is rotated 90 degrees counterclockwise, so the orientation marker is directed cephalad. With subtle adjustments and while not losing view of the right atrium, the IVC comes into focus with the RA-IVC junction on the screen. An ideal subcostal view also demonstrates the hepatic veins emptying into the IVC, along with the IVC emptying into the RA. 

## Measurements 

### Left Ventricular Systolic Function Assessment

Assessment of left ventricular systolic function includes calculation of the stroke volume and also ejection fraction. The steps involved in this assessment are as follows:

### Measurement of Left Ventricular Outflow Tract (LVOT)

Measurement of left ventricular outflow tract diameter is crucial for assessment of left ventricular systolic function. Once PLAX views are obtained, focus is directed towards the aortic valve and the LVOT to capture the maximal opening of the AV valve in mid-systole. The diameter of the LVOT at the base of the AV is measured. The LVOT area is calculated based on the equation below 8. 

LVOT area = π x (radius of LVOT)^2^

### Measurement of Velocity Time Integral (VTI)

Once apical 4-chamber views are obtained, with slight manipulation of technique, apical 5 chamber views can be obtained. Once this view is obtained, pulse wave doppler can be centered in the LVOT to obtain the LVOT systolic flow velocity. Once the systolic flow velocity is traced, the machine software can calculate the area under the curve which will results in estimation of the velocity time integral (VTI). The VTI (measured in centimeters) represents the distance blood is propelled forward from the LVOT to the aorta with each cardiac contraction. 

### Calculation of Stroke Volume 

Once the LVOT diameter and VTI have been entered into the ultrasound, the available software calculates the left ventricular stroke volume (LVSV) according to the equation: 

 LVSV (cm^3^) = LVOT Area (cm^2^) X LVOT VTI (cm) 

The formula is derived from the volume of a cylinder while considering that LVOT is the area of the cylinder and VTI represents the height of the cylinder, so the LVSV is a product of these two measurements. 

### EPSS (E-Point Septal Separation) 

**Figure 3:**
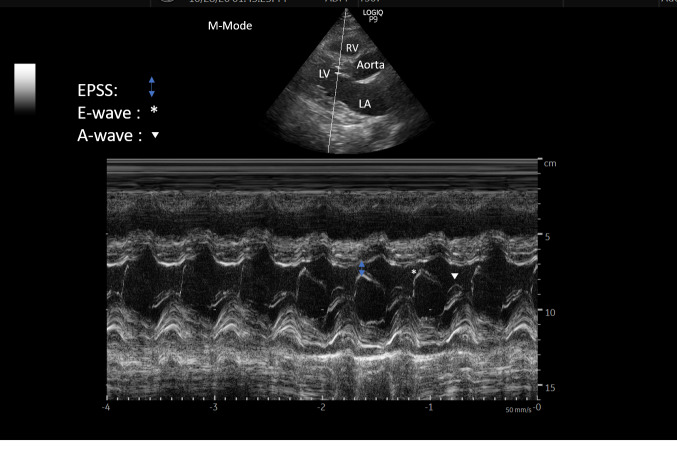
E point septal separation-definition and measurement.

The major chambers on the left side of the heart, the left atrium and left ventricle, are separated from each other by the mitral valve. The mitral valve is made up of two leaflets: anterormedial and posterolateral. The mitral valve is typically 4-6 cm^2^ in area. In the PLAX view, using the M-mode (motion mode), the cursor is placed through the mitral valve. In order of sequence (from top to the bottom) the RV, interventricular septum, anterior mitral valve leaflet, and posterior ventricular wall can be seen. The first peak (E-wave) noted during mitral valve tracing is the passive diastolic filling of the left ventricle while the following, smaller A-wave represents LV filling from LA contraction. EPSS represents the distance separating the peak height of the E-wave from the interventricular septum during early diastolic filling. EPSS can be thought of most simply as inversely correlated with left ventricle contractility and ejection fraction. A normal, healthy anterior MV leaflet may come in contact with the septum creating zero distance of E-point separation. An EPSS greater than 7 mm may be used to predict patients with severely reduced LVEF 9. The technique for measurement of EPSS is shown in Figure 3.

### Tricuspid Annular Plane Systolic Excursion (TAPSE)

A commonly used parameter for measurement of right ventricular function is called tricuspid annular plane systolic excursion (TAPSE).In an apical 4‑chamber view, the maximum systolic excursion of the lateral tricuspid annulus is measured by M‑mode. The tracing can be acquired easily. Though TAPSE varies from individual to individual based on gender and body surface area, a TAPSE < 17 mm is highly suggestive of RV dysfunction 10.

### Inferior Vena Cava Measurements (IVC Measurements)

A routine part of POCUS should include IVC measurements. Images are obtained as described above in the subcostal view of the heart. One important aspect to remember while acquiring images of the IVC is to obtain images longitudinally with the transducer centered on the long axis of the IVC to accurately estimate IVC diameter and collapsibility. Imaging outside the longitudinal axis of the IVC will result in oblique view of the vessel and underestimation of the vessel diameter in the so-called “cylinder effect”. Using M-Mode, the diameter of the IVC can be calculated. The IVC is subject to great degree of variation due to the effect of the RA. IVC diameter <2.1 cm that collapses >50% suggests normal RA pressure of 3 mm Hg (range, 0–5 mm Hg), whereas IVC diameter >2.1 cm that collapses <50% with a sniff suggests high RA pressure of 15 mm Hg (range, 10–20 mm Hg) 11. IVC measurement may not reliably predict right atrial pressure and volume status in patients who have intraabdominal hypertension, acute excerbation of airways disease such as asthma or COPD, right ventricular failure, or pericardial tamponade as well as those on positive pressure ventilation. Similarly, in conditions of severe tricuspid regurgitation and pericardial constriction, there is likely to be a discordance between IVC measurements and the actual volume status of a patient. Incorrect techniques of image acquisition, movement of the transducer while using M-mode and lateral sliding of the IVC relative to the transducer with inspiration may introduce additional sources of error that operators and interpreters must keep in mind 12.

## Clinical Application of FOCUS in Nephrology Practice

**Figure 4:**
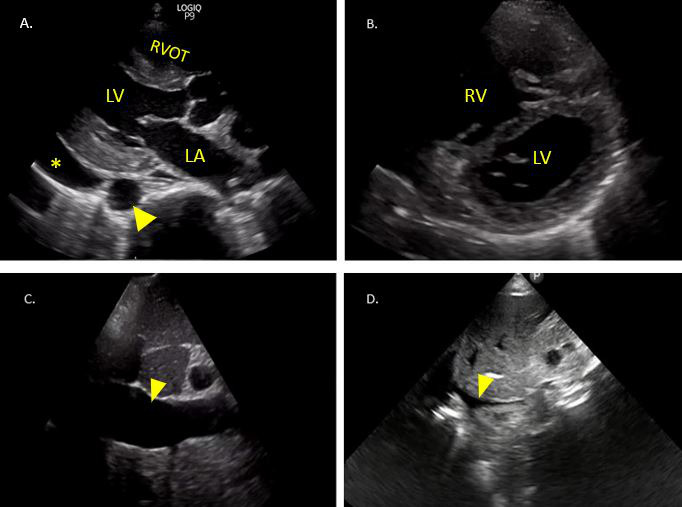
A) Pericardial effusion (*) in the PLAX view. Arrowhead denotes descending thoracic aorta. B) D-shaped LV in RV overload (interventricular septal flattening). C) Plethoric IVC (arrowhead). D)Collapsible IVC (arrowhead). LA - left atrium, LV - left ventricle, RV - right ventricle, RVOT - right ventricular outflow tract, IVC - inferior vena cava.

Emergency medicine programs use the 5E’s mnemonic to gather valuable information from FOCUS which includes effusion, ejection, equality, exit, and entrance. Applying this information to clinical nephrology can help in a variety of scenarios described below that are frequently encountered on the nephrology consult service and dialysis rounds. A few representative images of abnormalities that may be seen on FoCUS are depicted in Figure 4.

**Dyspnea:** Dyspnea is a common presenting complaint in patients with kidney disease 13. The differential for dyspnea is broad and includes, amongst other causes, LV dysfunction (leading to pulmonary edema), acute RV strain (in the setting of pulmonary embolism), valvular disorders (for example, mitral regurgitation), or pericardial effusion. Prompt bedside FoCUS can help delineate these different etiologies of dyspnea and guide further management as clinically indicated. 

**Hypotension**: Hypotension is frequently encountered in dialysis patients^2^, ^14^ and this is also a risk factor for development of hemodynamic acute kidney injury 15. Hypotension can also manifest in the different types of shock seen in critically sick patients which may be challenging to differentiate clinically. FoCUS can help rapidly identify the etiology of shock as cardiac versus non-cardiac and guide initiation of appropriate protocols for treatment in a timely and efficient manner. 

**Volume management:** The need for volume assessment and assistance with management is a frequent reason for requesting nephrology consultation in the hospital. IVC assessment has been shown to correlate well with RAP 11. The caveat here is that IVC assessment needs to be interpreted in the appropriate clinical context and may not provide accurate estimation of the volume status in some scenarios including mechanically ventilated patients^11^ and those with intraabdominal hypertension 16. Serial measurements of the IVC can help in guiding diuretic management in fluid overloaded patients. Also, IVC assessment in dialysis patients can help add precision to adjustment to ultrafiltration goals and estimated dry weight with dialysis 17.

In conclusion, FoCUS is an attractive modality for the busy nephrologist with the ability to provide quick, point of care answers to common clinical questions. 

## Disclosures

The authors have no conflicts of interest to disclose. No funding was obtained for this work. 
